# SiamEFT: adaptive-time feature extraction hybrid network for RGBE multi-domain object tracking

**DOI:** 10.3389/fnins.2024.1453419

**Published:** 2024-08-08

**Authors:** Shuqi Liu, Gang Wang, Yong Song, Jinxiang Huang, Yiqian Huang, Ya Zhou, Shiqiang Wang

**Affiliations:** ^1^School of Optics and Photonics, Beijing Institute of Technology, Beijing, China; ^2^Center of Brain Sciences, Beijing Institute of Basic Medical Sciencesy, Beijing, China

**Keywords:** RGB and Event, spatio-temporal, hybrid network, spiking neural networks, neuromorphic computing, object tracking

## Abstract

Integrating RGB and Event (RGBE) multi-domain information obtained by high-dynamic-range and temporal-resolution event cameras has been considered an effective scheme for robust object tracking. However, existing RGBE tracking methods have overlooked the unique spatio-temporal features over different domains, leading to object tracking failure and inefficiency, especally for objects against complex backgrounds. To address this problem, we propose a novel tracker based on adaptive-time feature extraction hybrid networks, namely Siamese Event Frame Tracker (SiamEFT), which focuses on the effective representation and utilization of the diverse spatio-temporal features of RGBE. We first design an adaptive-time attention module to aggregate event data into frames based on adaptive-time weights to enhance information representation. Subsequently, the SiamEF module and cross-network fusion module combining artificial neural networks and spiking neural networks hybrid network are designed to effectively extract and fuse the spatio-temporal features of RGBE. Extensive experiments on two RGBE datasets (VisEvent and COESOT) show that the SiamEFT achieves a success rate of 0.456 and 0.574, outperforming the state-of-the-art competing methods and exhibiting a 2.3-fold enhancement in efficiency. These results validate the superior accuracy and efficiency of SiamEFT in diverse and challenging scenes.

## 1 Introduction

Visual object tracking is a significant research area within computer vision, which is to continuously estimate the location and size of an object in subsequent frames based on the bounding box provided for the initial frame. Artificial neural networks (ANNs) have good performance in general visual object tracking (Jiao et al., 2021) due to substantial learning capabilities. However, in challenging and complex scenes, such as low illumination or fast motion (Huang et al., 2019), the accuracy and robustness of the trackers are compromised. The primary limitation arises from the tracking datasets used in the networks, which consist of visible images captured by traditional frame-based cameras (Boettiger, 2021). These images have limited frame rates and dynamic ranges, and lack time information with high temporal resolution and spatial information beyond the dynamic range. Therefore, employing an auxiliary modality for tracking has emerged as a prevalent approach to address these limitations. Numerous studies have attempted to enhance tracking performance by leveraging the complementary information from thermal and depth modalities, such as RGBT tracking (Li et al., 2020; Lu et al., 2021; Wang F. et al., 2023; Zhao et al., 2023) and RGBD tracking (Liu et al., 2020; Wang et al., 2020; Yan S. et al., 2021; Yang et al., 2023). However, these trackers fail to yield satisfactory results in scenes involving high dynamic range and fast motion.

Event-based cameras (Gallego et al., 2020) are bio-inspired, which simulate the coding mechanism of lower animal retina to dynamic objects. The high dynamic range of event cameras allows for the depiction of fast-moving objects under diverse lighting conditions. Nevertheless, events are only triggered when brightness changes surpass a certain threshold, thus the obtained events are unable to represent absolute light intensity. Visible images provide abundant spatial texture details of objects, while events deliver temporal data unaffected by the motion blur of the object and edge details unaffected by poor lighting conditions. The integration of visible images and events provides a more comprehensive representation of objects information. Some studies have utilized ANNs to integrate RGB and event data for developing multi-domain trackers (Zhang et al., 2021a; Wang X. et al., 2023; Zhang J. et al., 2023). However, these methods fail to effectively extract information from multi-domain and do not achieve a balance between speed and accuracy.

ANNs are proficient in extracting spatial features from visible data but face challenges in capturing temporal features from asynchronous input event data, resulting in diminished accuracy in object tracking. In contrast, spiking neural networks (SNNs) are adept at processing event data and exhibit strong capabilities in extracting temporal features. Inspired by biological mechanisms (Wu et al., 2018; Chakraborty et al., 2021; Niu et al., 2023), SNNs provide significant computational power with minimal energy consumption by simulating the function of biological neurons to process binary event data in multiple time steps (Roy et al., 2019; Xu et al., 2022; Zhang H. et al., 2023). Consequently, integrating SNNs with ANNs in RGBE multi-domain object tracking improves both accuracy and efficiency (Yang et al., 2019; Zhang et al., 2021b; Zhao et al., 2022).

In this paper, we propose the SiamEFT (Siamese Event Frame Tracker), designed to improve the accuracy and efficiency of tracking fast-moving objects in complex scenes. The tracker proficiently integrates RGB domain feature extracted by ANNs with event domain feature extracted by SNNs. First, we transform the SiamRPN++ (Li et al., 2019) framework by substituting LIF neurons (Hunsberger and Eliasmith, 2015) for ANN neurons to an SNN version as the backbone of SiamEFT. Then, the cross-network fusion module (CNF) combines spatio-temporal feature information from multi-domain to facilitate object tracking. Further, during the data preprocessing phase, we use the adaptive-time Attention module (ATA) to effectively incorporate crucial object information from the event flow while eliminating redundant data. This tackles the issue of sparse and irregular event flow, leading to improved accuracy and efficiency in tracking fast-moving objects within complex scenes. To sum up, the major contributions are as follows:

- We introduce an adaptive-time attention module (ATA) to consolidate event flow into frames, enhancing the representation capability of event data and boosting the tracking efficiency of the network.
- We propose a Siamese-based multi-domain feature extraction hybrid network **SiamEFT**, which employs the cross-network fusion module (CNF) to effectively merge spatio-temporal information from both the frame and event domains.
- Extensive experiments on two realistic RGBE tracking benchmarks demonstrate the superior performance of our SiamEFT in comparison to state-of-the-art methods.

## 2 Related work

RGB and Event (RGBE) multi-domain object tracking methods are roughly divided into traditional and deep learning methods. In traditional methods, Huang et al. (2018) identified patches of Canny edges within grayscale frames and tracked these local edge patterns across the event stream. Gehrig et al. (2020) using the method of raw intensity measurement utilized frame-based feature extraction and used events to track asynchronously. Traditional methods depend on manually designed strategies, which frequently require tedious fine-tuning across various application scenarios. With the advancements in deep learning (LeCun et al., 2015), neural networks have been increasingly utilized for RGBE tracking, which can be categorized into two primary methods. One is using only ANNs, and the other is combining ANNs and SNNs.

### 2.1 ANNs for RGBE tracking

Currently, ANNs serve as the primary approach for RGBE tracking. Zhang J. et al. (2023) proposed the AFNet, a Siamese network comprising multi-modal alignment and fusion modules. By incorporating an event-guided cross-modal alignment module and a cross-correlation fusion module, this tracker integrated and aligned visible and event data to achieve high frame rate object tracking. However, there remains potential to further enhance its tracking accuracy. Wang X. et al. (2023) proposed the CMT-MDNet model, employing a convolutional neural network for feature extraction and utilizing a cross-modality transformer module for interactive feature learning and fusion, thereby enhancing the fusion of visible data and event data for precise object tracking. Although this method enhances tracking accuracy, it may neglect considerations of efficiency.

### 2.2 ANNs and SNNs for RGBE tracking

ANNs have achieved notable success in object tracking tasks with traditional frame-based cameras. However, they face challenges in efficiently process event data captured by event cameras. SNNs offer distinctive capabilities for asynchronous event data processing. Chakraborty et al. (2021) proposed a neuronal model based on synaptic scaling mechanism and applied it to spiking neural networks, which has significant advantages in RGB or Event single-domain object detection task. Xu et al. (2022) proposed a fully spiking neural network, which uses STDP and back-propagation hybrid learning methods to train and encodes RGB domain datasets into spiking sequences during inference, thus achieving energy-efficient object detection. The above studies highlight that SNNs offer significant advantages in processing event data domain, particularly in efficiency and energy savings.

Based on the unique characteristics of ANNs and SNNs, several studies consider combining the advantages of the two to apply in different fields to further improve the performance. For example, Lee et al. (2020) presented a deep hybrid neural network integrating SNNs and ANNs for efficiently estimating optical flow from sparse event camera outputs, thus providing significant computational efficiency. Due to the sparse and irregular nature of event data, object information may not be accurately provided in some complex scenes, which will affect the accuracy of the network. Therefore, it is essential to integrate RGB and Event multi-domain to more comprehensively capture and express the object information.

Consequently, several studies have attempted to combine ANNs and SNNs for RGBE object tracking, aiming to improve both tracking accuracy and efficiency. Yang et al. (2019) proposed the DashNet, which employs a time complementary filter and an attention mechanism module to fuse multi-domain features processed by ANNs and SNNs, thereby demonstrating the advantages of combining the two networks in a single model. Zhao et al. (2022) designed a hybrid neural network and proposed a hybrid unit as a link interface between ANNs and SNNs, promoting the cross-paradigm modeling of general artificial intelligence. These studies have promoted the design of hybrid networks and provided an effective and efficient solution for object tracking. The evaluation of these studies is for some real scenes or simulated data, which complicates the assurance of tracking performance in complex practical scenes such as high-speed motion or environments with a wide dynamic range. Zhang et al. (2021b) introduced the MCFR, which includes a feature extractor designed around ANNs and SNNs to isolate unique and common features from the visible and event domains respectively.This method pays more attention to using complementary information of different domain to improve tracking accuracy, and may ignore the ability of different networks to extract unique spatial features and temporal features.

## 3 Methodology

### 3.1 Overview of network structure

Our methodology is predicated on two crucial observations. First, the integration of spatially rich information from RGB with temporally detailed data and edge information from events facilitates a more comprehensive representation of object information. Secondly, ANNs excels at extracting distinct spatial features from RGB images, whereas SNNs effectively captures unique temporal features from event data (Di Caterina et al., 2024). Consequently, effective fusion of spatio-temporal information from both networks is crucial for improving tracking accuracy and efficiency in practical scenes with high dynamic range backgrounds and fast-moving objects.

**Figure 1:**
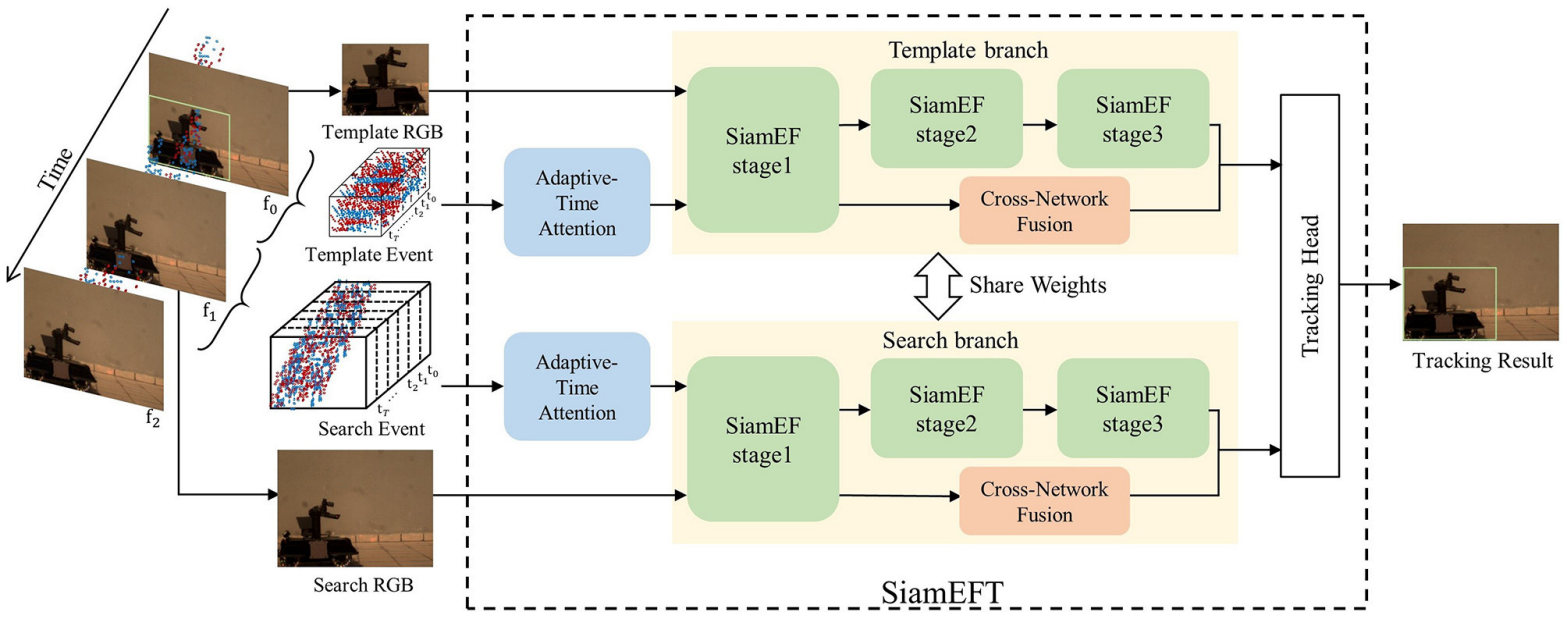
Our SiamEFT pipeline consists of three primary components: ATA utilizes adaptive weights to adjust event frames, SiamEF incorporates ANNs and SNNs to extract multi-domain information, and CNF performs cross-network information fusion. The final output is generated by the head module. The object is a high-speed toy truck reproduced in the VisEvent dataset.

As shown in Figure 1, the SiamEFT is composed of three main components: the data preprocessing module, the SiamEF module, and the head module. In the data preprocessing module, the adaptive-time attention module (ATA) is used to adjust the weight of the T event frames for the input template event and search event. Following the Siamese network (Zhou and Zhang, 2022), the SiamEF consists of a template branch and a search branch with shared weights. In each branch, RGB data is processed in three stages within SiamEF to extract deep features, while event images are processed in the initial stage of the SiamEF to extract event information. Subsequently, the cross-network fusion module (CNF) is utilized for multi-modal fusion. In the head module, the response map and candidate bounding box are generated through two distinct branches, which collectively calculate the final tracking outcome.

### 3.2 Event representation

Unlike frame-based cameras, event-based cameras capture asynchronous event streams of logarithmic-scale brightness changes per pixel, providing remarkably wide dynamic ranges and exceptional temporal resolution in the microsecond range. The event stream ε comprises *N* event data points, each represented by a quadruple and is expressed as Equation 1:


(1) $$\begin{array}{l} \varepsilon ={\left[e_{k}\right]}_{k=1}^{N}={\left[\left\{x_{k},y_{k},p_{k},t_{k}\right\}\right]}_{k=1}^{N} \end{array}$$

where *e*_*k*_ denotes the *k*-th event, (*x*_*k*_, *y*_*k*_) is the pixel position of *e*_*k*_, *t*_*k*_ is the timestamp when the event is triggered, *p*_*k*_ ∈ {+1, −1} is the polarity: “+1” denotes the brightness enhancement at the pixel point and “1” denotes the brightness reduction.

To extract pertinent information from the event stream effectively, it is crucial to tailor event data expression forms to specific tasks (Sekikawa et al., 2019; Shi and Rajkumar, 2020; Wang et al., 2021). For RGBE object tracking (Pérez-Carrasco et al., 2013), bridging the domain gap between RGB and event data requires adopting a grid-based representation of events similar to RGB images. For each frame-based image *F*_*i*_ at the timestamp *t*_*i*_, the event stream is corresponding stacked at intervals of [*t*_*i*_−Δ*t, t*_*i*_ + Δ*t*) and *T* event frames with time resolution of *dt* corresponding to *F*_*i*_ are obtained to form the event frame group *E*_*i*_. The pixel value of each event frame in *E*_*i*_ represents the cumulative aggregation of the polarity of the event at the pixel during the *dt* period.

The process to construct the event frame group *E*_*i*_ is described by the Equation 2:


(2) $$\begin{array}{l} \left\{ \begin{array}{l} j_{l}=t_{i}-\text{Δ}t+dt\times j\\ j_{r}=t_{i}-\text{Δ}t+dt\times (j+1)\\ E_{i}(j,p,x,y)=[\sum_{t_{i}=j_{l}}^{j_{r}}{ℶ}_{p,x,y}(p_{i},x_{i},y_{i}){]}_{j=0}^{T-1} \end{array}\right. \end{array}$$

where ℶ is a characteristic function: when *p, x, y* = (*p*_*i*_, *x*_*i*_, *y*_*i*_), the value is 1; otherwise, it is 0.

### 3.3 Adaptive-time attention module for event feature extraction

The event flow is marked by its sparsity and non-uniformity (Gallego et al., 2020), triggered exclusively when the change in light intensity at a pixel exceeds a predefined threshold. When the light intensity in the tracked environment slightly changes, the event stream is sparse, leading to the accumulation of event frames may contain blank or minimally informative frames. Conversely, in environments characterized by frequent changes in light intensity, the accumulated event frames contain more information.

Given the distinctive characteristics of event streams, preprocessing the generated event frames is crucial for maximizing the utilization of event data and enhancing the efficiency of network information processing. The adaptive-time attention module (ATA) is designed to extract pertinent information and eliminate redundant information from event frames and the primary objective is to estimate the weight score of each frame within the *T* event frames. The weight scores are related to both the spatial features of the current time step and the spatio-temporal information from adjacent event frames.

**Figure 2:**
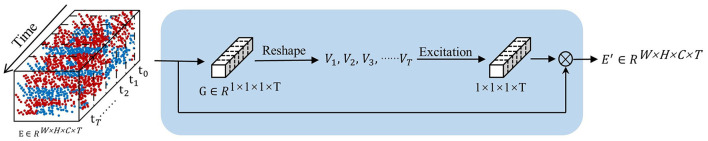
Adaptive-time attention module. The size of the T event frames E remains unaltered following this module, while the spatial information depicted in each frame is assigned an adaptive weight based on the time information dimension.

As shown in Figure 2, the global spatial feature vector of each event frame is obtained on the *T* event frames sorted by time. The feature vector *G*^*t*^ obtained from the *t*-th event frame *E*^*t*^∈*R*^*W*×*H*×*C*^ can be expressed as Equation 3:


(3) $$\begin{array}{l} G^{t}=\frac{1}{W\times H\times C}\overset{C}{\underset{c=1}{\sum }}\overset{W}{\underset{w=1}{\sum }}\overset{H}{\underset{h=1}{\sum }}E^{t}\left(c,w,h\right) \end{array}$$

Upon obtaining all the global spatial feature vectors *G* from the *T* event frames sequence, reshaping *G* sequence yields the time information vector *V*. *V* is utilized in the excitation operation (Hu et al., 2018) to establish correlations in the time dimension, thereby determining the weight score for each frame. Finally, the weight score multiplied by the original *T* event frames executes adaptive time calibration.

### 3.4 The SiamEF module and cross-network fusion module

**Figure 3:**
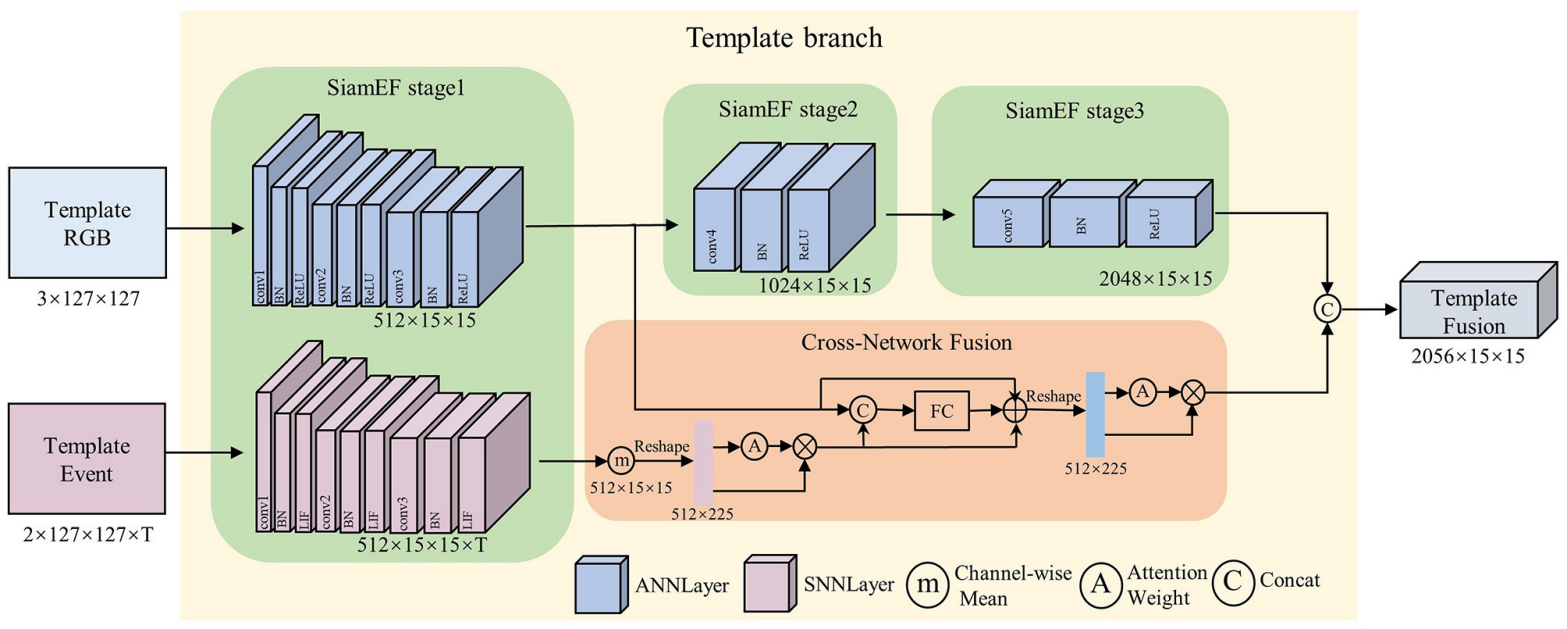
The SiamEF module uses ANN for feature extraction from the RGB domain and SNN for feature extraction from the event domain. The CNF module is then used to fuse the extracted spatio-temporal information.

The feature extraction stage of the Siamese network comprises two branches: the template branch and the search branch, both of which have identical network architectures and share weight. As illustrated in Figure 3, the template branch serves as an exemplar to show the feature extraction process of RGB and event multi-domain in the SiamEF module and the integration process of the cross-network fusion module.

The SiamEF module employs ANNs and SNNs to extract feature information from the RGB and event domains, respectively. ANNs uses ResNet50 (He et al., 2016) as the backbone to extract in-depth spatial features from the RGB domain, capturing and preserving features across the third, fourth, and fifth layers of the output in all three stages of SiamEF. SNNs adopts LIF neurons instead of neurons in ResNet50-2stage architecture to extract spatio-temporal information from the event domain. Selective employment of ResNet50-2stage architecture significantly reduces network parameters, thereby enhancing processing speed while preserving the accuracy of results. Ultimately, the SiamEF module generates multi-domain feature maps.

In the cross-network fusion module, the feature maps from the RGB and event domains acquired in the first stage of the SiamEF module are fused. Given the input RGB feature map *F*_*R*_ and Event feature map *F*_*E*_, *F*_*E*_ is averaged along on the T-channel dimension to ensure the size aligns with the RGB feature. Subsequently, the spatio-temporal information in *F*_*E*_ are optimized to obtain $F_{E}^{\prime }$, defined as Equation 4:


(4) $$\begin{array}{l} F_{E}^{\prime }=\text{A}\left(R^{\left(\left(C,WH\right)\right)}\left(F_{E}\right)\cdot R^{\left(\left(WH,C\right)\right)}\left(F_{E}\right)\right)\times F_{E} \end{array}$$

where $R^{\left(\left(\cdot \right)\right)}$ denotes reshape function with object shape (·), A denotes the softmax of the obtained spatio-temporal attention weights.

The optimized $F_{E}^{\prime }$ is integrated with *F*_*R*_ representing strong spatial information to obtain *F*_*M*_ for further enhancing the discriminative spatial features. The formula is as Equation 5:


(5) $$\begin{array}{l} F_{M}=L\left(C\left(\left[F_{R},F_{E}^{\prime }\right]\right)\right)+F_{R}+F_{E}^{\prime } \end{array}$$

where C represents the concatenation of *F*_*R*_ and $F_{E}^{\prime }$, L represents the mapping of features.

The spatio-temporal information of the *F*_*M*_ is further optimized, yielding a final fused feature map enriched with comprehensive spatio-temporal details. Subsequently, the final tracking result is obtained by inputting this fused feature map into the head component.

## 4 Experiments

### 4.1 Experimental setting

#### 4.1.1 Datasets

We evaluate the proposed SiamEFT on two realistic large-scale RGBE tracking benchmarks **VisEvent** (Wang X. et al., 2023) and **COESOT** (Tang et al., 2022), which include the RGB data in the multiple scenes and the event data aligned in the same scenes. VisEvent comprises 709 short-term and 111 long-term tracking sequences across 17 challenging real-world scenes, including low light, high dynamic range, fast motion, and motion blur. COESOT employs a zoom lens camera to capture videos and contains numerous scale-changing scenes. It is the most large-scale and modal-aligned dataset, comprising over 1,300 video sequences.

#### 4.1.2 Evaluation metric

The evaluation of tracking performance includes quantitative and qualitative evaluations. We adopt two widely-used tracking metrics, the **success rate** (SR) and the **precision rate** (PR) to quantitatively assess the performance of tracker. SR measures the accuracy of size and scale by calculating the percentage of frames where the overlap between the predicted and ground-truth bounding boxes exceeds a predetermined threshold. PR measures positioning accuracy by calculating the proportion of frames in which the distance between the centers of the predicted and ground-truth bounding boxes falls within a predetermined threshold. We calculate the area under the curve as the representative SR (RSR) and the PR score related to the *20-pixels* threshold (Wang X. et al., 2023) as the representative PR (RPR).

#### 4.1.3 Experiments details

In the training process, we utilize the SiamRPN++ (Li et al., 2019) within ANNs framework as baseline, and train RGB domain data to obtain the initial weight. Subsequently, using ANN-TO-SNN training method the ReLU function is replaced by LIF neurons and expanded to SNN variant, resulting in the SiamEFT that integrates both ANNs and SNNs. The initial weight is then shared to generate the complete weight files. Finally, RGB and event multi-domain data are input into the SiamEFT to retrain again based on the above complete weight.

Based on Python 3.8 and PyTorch 1.2 (Paszke et al., 2019) deep learning API, we choose AdamW (Kingma and Ba, 2014) as the optimizer, with an NVIDIA RTX 4060 Ti GPU across 50 epoches with 152,600 samples per epoch. We set the initial learning rate to 1e-3 with a warm-up period also at this rate. During the multi-domain training process, the ANNs branch is frozen until the 20-th epoch to ensure stability in the training process. The batch size is 32, the input template RGB image size is 127 × 127 × 3 and the search RGB image is 271 × 271 × 3. We integrate the event data with 10 time steps per sample using SpikingJelly (Fang et al., 2023) package.

To comprehensively verify the performance of our tracker, we compare our tracker with seven competitive methods, including CF-based [ATOM (Danelljan et al., 2019), SuperDiMP (Bhat et al., 2019), AFNet (Zhang J. et al., 2023)], Siamese-based [SiamRPN (Li et al., 2018)], Transformer-based [STARK-ST101 (Yan B. et al., 2021), Mixformer22k (Cui et al., 2022)], and Multi-Domain-based [CMT-MDNet (Wang X. et al., 2023)] trackers. The event representations of all trackers are the same as our trackers for fair comparison.

### 4.2 Quantitative evaluation

#### 4.2.1 Overall performance

**Figure 4:**
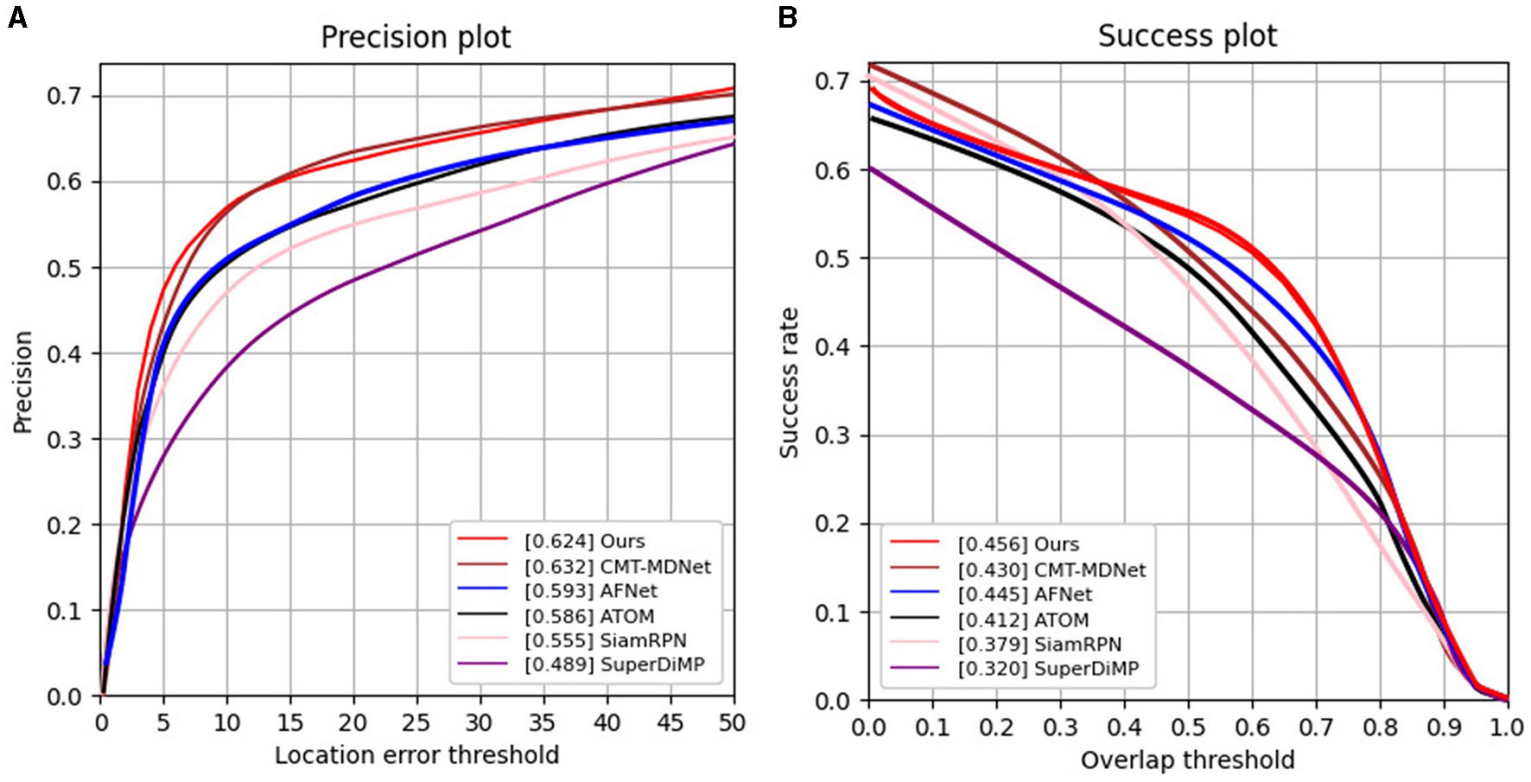
PR and SR curves of different tracking results on the VisEvent dataset. **(A)** Results of precision rate evaluation; **(B)** Results of success rate evaluation.

**VisEvent**. First, we compare our SiamEFT with other five trackers on the VisEvent dataset, namely CMT-MDNet (Wang X. et al., 2023), AFNet (Zhang J. et al., 2023), ATOM (Danelljan et al., 2019), SiamRPN (Li et al., 2018), and SuperDiMp (Bhat et al., 2019). The comparison results are shown in Figure 4. In particular, our SiamEFT (62.4%/45.6% in PR/SR) outperforms 2.6% over the state-of-the-art CMT-MDNet in SR, and is close to the CMT-MDNet in PR.

**Figure 5:**
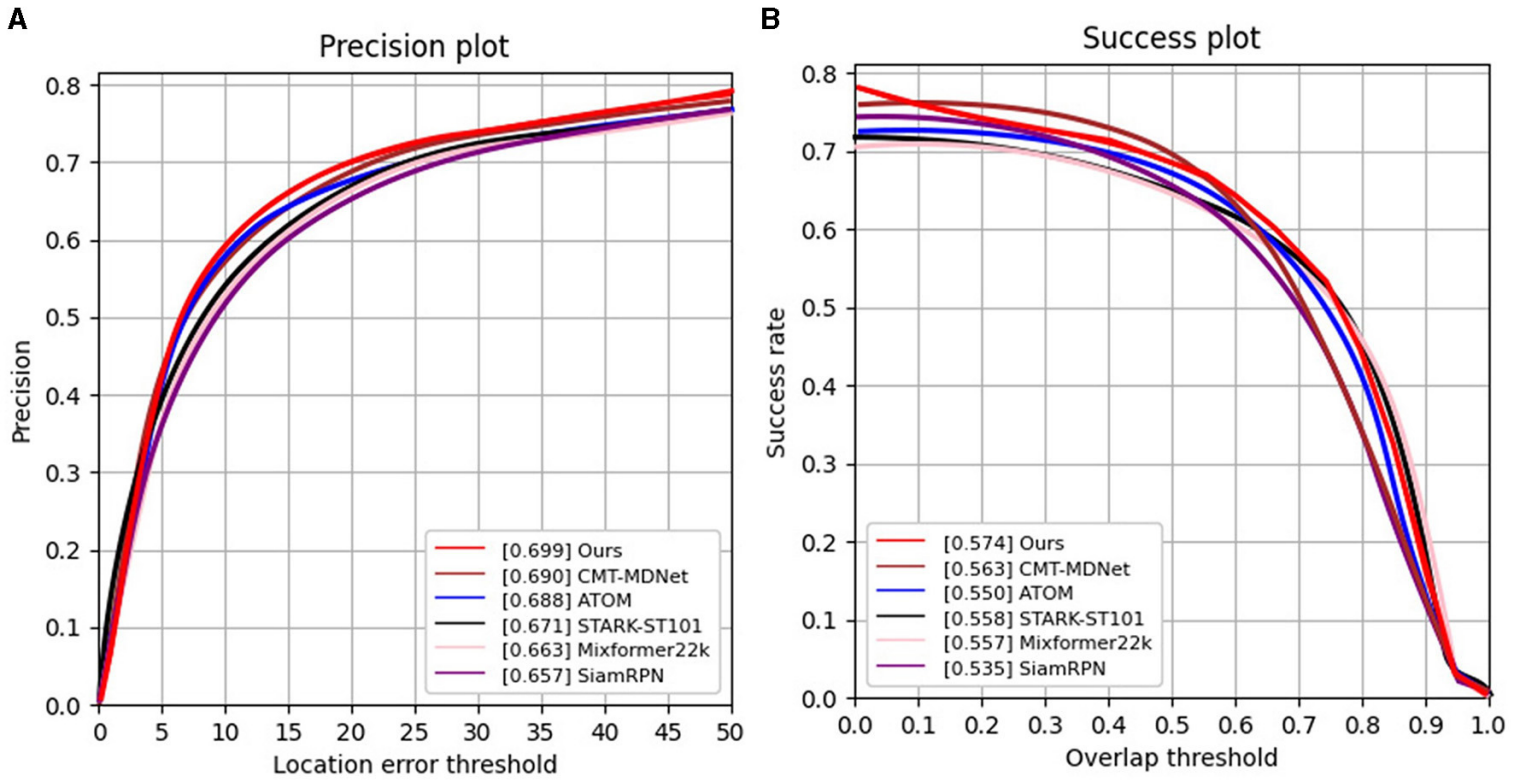
PR and SR curves of different tracking results on the COESOT dataset. **(A)** Results of precision rate evaluation; **(B)** Results of success rate evaluation.

**COESOT**. Second, we evaluate our SiamEFT with competitive trackers on the COESOT dataset, namely CMT-MDNet (Wang X. et al., 2023), ATOM (Danelljan et al., 2019), STARK-ST101 (Yan B. et al., 2021), Mixformer22K (Cui et al., 2022), and SiamRPN (Li et al., 2018). Given the diverse challenging conditions prevalent in both the RGB and event domains, the objects in COESOT display greater size variability compared to those in VisEvent. As shown in Figure 5, our SiamEFT achieves PR and SR of 69.9% and 57.4%, respectively, surpassing the other five evaluated trackers. It outperforms the most advanced tracker by 0.9% in PR and 1.1% in SR.

#### 4.2.2 Accuracy vs. speed

We evaluate the balance speed performance of our SiamEFT against the most representative trackers of different types on VisEvent. When calculating the frame rate using only RGB frames, as shown in Table 1, our SiamEFT achieves 31.6 FPS, significantly surpassing the tracking rate of the most accurate tracker CMT-MDNet 14 FPS. Moreover, it reaches an impressive 109 FPS in the combined RGB and event multi-domain. Our SiamEFT enhances the tracking efficiency significantly while maintaining high accuracy.

**Table 1:** Overall tracking performance on VisEvent.

**Trackers**	**SiamEFT**	**CMT-MDNet**	**ATOM**	**SiamRPN**
RPR	0.624	0.632	0.586	0.555
RSR	0.456	0.430	0.412	0.379
Speed (FPS)	31.6	14	30	28.5

### 4.3 Quantitative evaluation

**Figure 6:**
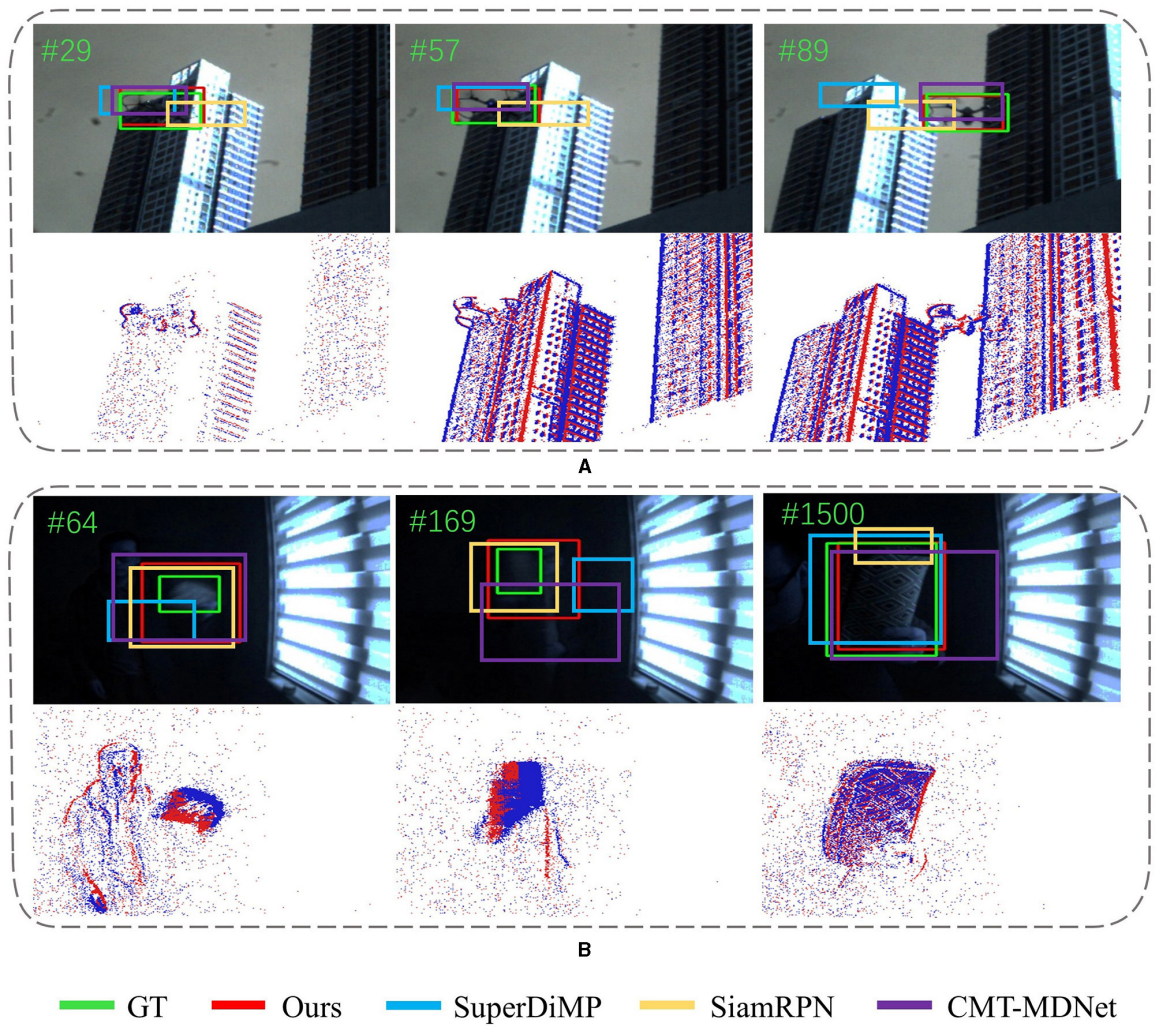
Qualitative evaluation of SiamEFT against other state-of-the-art trackers on various challenges in both RGB and event modalities. **(A)** The UAV is in fast motion. **(B)** The book is in low illumination.

We compare our tracker with state-of-the-art trackers under four different challenging conditions including fast motion, low illumination, minimal object, and high dynamic range. In Figure 6, the first row visually compares the tracking results in the RGB domain under conditions of fast motion or low illumination, and SiamEFT can accurately track the objects. The second row visualizes the representations in the corresponding event domain, and reveal the event frame exhibits the clearer objects edge representation compared to the RGB frames.

**Figure 7:**
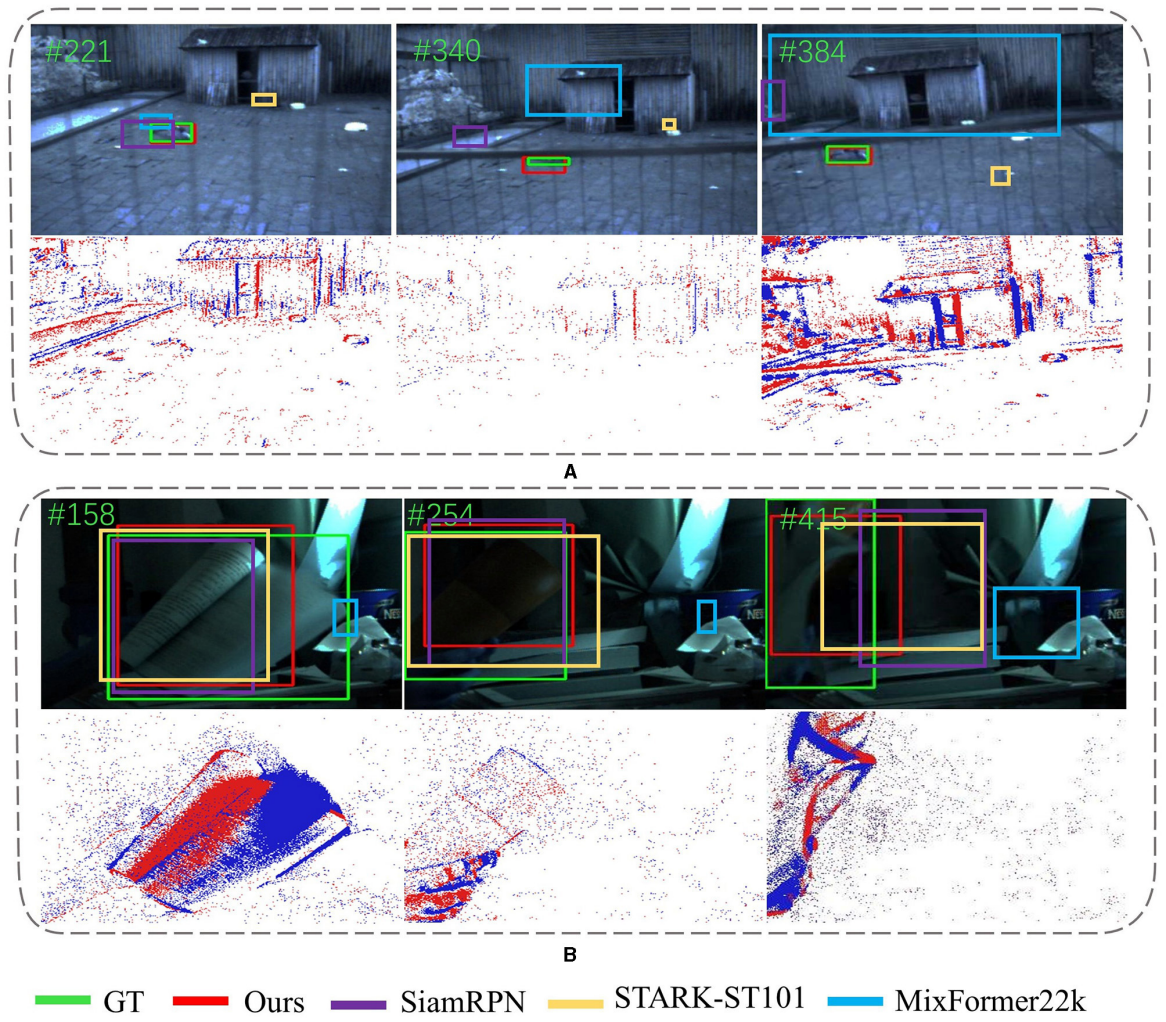
Qualitative evaluation of SiamEFT against other state-of-the-art trackers on various challenges. **(A)** The animal is a tiny target. **(B)** The book is in high dynamic range.

In addition, as shown in Figure 7, our SiamEFT continutes to track objects accurately in environments with high dynamic range or when the objects are exceedingly tiny. Notably, Figure 7A presents a particularly challenging scene where the tracking object is tiny and the background is complex. Upon these conditions, the event data struggles to effectively delineate the contour. Consequently, object tracking primarily relies on the spatial feature information of the RGB domain processed by the ANNs. In Figure 7B, due to significant lighting changes, the RGB domain is not sufficient to represent spatial information such as color and texture associated with the object and tracking relies on the spatio-temporal of the event domain processed by SNNs. Hence, the combined capabilities of ANNs and SNNs in SiamEFT enable robust extraction and fusion of crucial object information, which facilitates precision and efficiency object tracking in complex scenes.

### 4.4 Ablation studies

To verify that the integration of RGB and event domains can improve the tracker performance, we implemented three variants: one using only RGB data as input, one using only event data as input (Chae et al., 2021) and one using both RGB and event data as inputs. The comparison results are shown in Table 2. The results demonstrate that the collaborative utilization of multi-domain information significantly outperforms the utilization of information from a single domain.

**Table 2:** Ablation study of SiamEFT on each module.

**Input domain**	**Network**	**Fusion strategy**	**ATA Module**	**VisEvent**	
				**SR**	**PR**
RGB	ANN	-		0.291	0.384
Event	ANN	-		0.252	0.372
RGB+Event	ANN	Merge		0.410	0.576
RGB+Event	SiamEFT	Merge		0.432	0.503
RGB+Event	SiamEFT	Concatenate		0.407	0.500
RGB+Event	SiamEFT	CNF module		0.436	0.595
RGB+Event	SiamEFT	CNF module	✓	0.456	0.624

✓ denotes that modules are deployed. For SR and PR: larger is better; the best performance as red; the second best as blue.

To evaluate the performance of the proposed CNF in extracting both common and unique features from the RGB and event domains, we developed three variants based on the SiamEF network. These variants include using merge to add the corresponding values of feature maps, using concatenate to stitch feature maps, and using CNF module to fuse feature maps. The results show that our CNF is superior to others, which indicates that SiamEFT with CNF can more effectively fuse different feature information obtained from RGB and event domians, thereby improving tracking performance.

To assess the effectiveness of the proposed ATA in extracting valuable information from event frames, we developed two variants: one is only stacking event data, the other is using ATA. The results demonstrate that the employment of ATA optimizes the information processing, thereby significantly enhancing tracking performance.

It is noteworthy that to use of the temporal information from event data more effectively, we refer to Danelljan et al. (2019) to set 10 time steps, which inevitably introduces latency to the network. When we set the time steps to 1, the SR / PR of the tracker is 42.8%/50.8% at a speed of 32 FPS. Setting the 10 time steps and utilizing the ATA module results in optimal accuracy improvements. The SR/PR is enhanced by 0.28%/11.6%, while maintaining a speed of 31.6 FPS with an average delay of just 0.4 millisecond per frame. Experiments demonstrate that introducing the time window and the design of ATA module enhance the network's accuracy and the delay of network is acceptable.

### 4.5 Failure cases analysis

**Figure 8:**
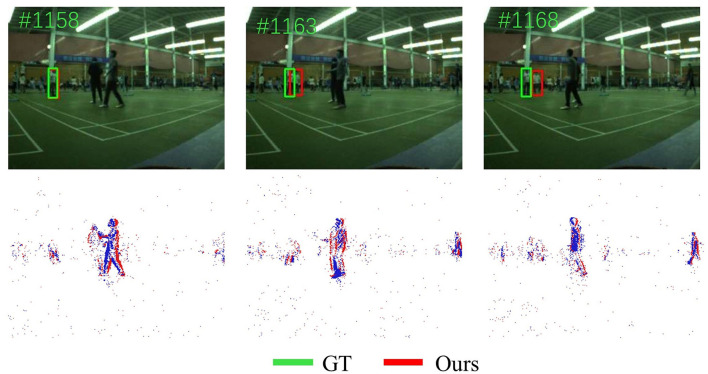
Visualization of failure cases of our SiamEFT. The green box represents ground truth, and the red box represents our SiamEFT tracking results.

Although this work achieves good results on some videos of dataset, our tracker also has some failures. As shown in Figure 8, SiamEFT will fail when the object closely resembles distractors and the distractor obstructs the object partially or completely. In our future work, we will consider enhancing the anti-interference mechanism in the tracker to better capture the spatio-temporal information of object tracking.

## 5 Conclusion

In this paper, we propose the SiamEFT effectively extract and integrate spatio-temporal features from RGB and event domains. This method addresses the challenges associated with inadequate extraction of spatio-temporal information in multi-domain contexts, thereby enhancing the precision and efficiency of object tracking. Specifically, the ATA module aggregates event data into frames using adaptive weights. Furthermore, we develop the SiamEF module, which leverages both ANNs and SNNs to extract features from both RGB and event domains. Finally, the CNF module is employed to effectively integrate the extracted spatio-temporal features. Extensive experimental evaluations on public RGBE datasets demonstrate that superior accuracy and efficiency of the proposed tracking method, especially in the case of low illumination or fast motion.In future work, we consider enhancing the anti-interference performance of the tracker to achieve higher tracking performance.

## Data availability statement

The datasets presented in this study can be found in online repositories. The names of the repository/repositories and accession number(s) can be found in the article/supplementary material.

## Author contributions

SL: Writing – original draft, Visualization, Software, Methodology, Conceptualization. GW: Writing – review & editing, Supervision, Project administration. YS: Writing – review & editing, Resources, Funding acquisition. JH: Writing – review & editing, Investigation. YH: Writing – review & editing, Visualization. YZ: Writing – review & editing. SW: Writing – review & editing.
